# ENAM Gene Variation in Students Exposed to Different Fluoride Concentrations

**DOI:** 10.3390/ijerph17061832

**Published:** 2020-03-12

**Authors:** Denisse Duran-Merino, Nelly Molina-Frechero, Leonor Sánchez-Pérez, Martina Nevárez-Rascón, Rogelio González-González, Omar Tremillo-Maldonado, Diana Cassi, Ronell Bologna-Molina

**Affiliations:** 1 Dental Sciences, Autonomous Metropolitan University Xochimilco (UAM), Calzada del Hueso 1100, Mexico City 04900, Mexico; duranmde7@gmail.com; 2 Department of Health Care, Autonomous Metropolitan University Xochimilco (UAM), Calzada del Hueso 1100, Mexico City 04900, Mexico; tlsperez@correo.xoc.uam.mx; 3 School of Dentistry, Autonomous University of Chihuahua (UACH), Chihuahua, Campus I Av. Universidad s/n, Chihuahua 31000, Mexico; martina.nevarez@gmail.com; 4 Department of Research, School of Dentistry, Juarez University of the State of Durango, Durango (UJED) Predio Canoas s/n, Durango 34000, Mexico; rogegg@hotmail.com (R.G.-G.); oatm88@gmail.com (O.T.-M.); 5 Department of Surgical, Medical, Dental and Morphological Science–University of Modena, 41121 Modena, Italy; dianacassi3@gmail.com; 6 Molecular Pathology Area, School of Dentistry, University of the Republic, Uruguay (UDELAR) Montevideo 11600, Montevideo 11200, Uruguay; ronellbologna@hotmail.com

**Keywords:** genetic variation, enamelin, dental fluorosis, caries

## Abstract

The ENAM gene is important in the formation of tooth enamel; an alteration can affect the lengthening of the crystals, and the thickness in enamel. The objective was to determine the presence of the single nucleotide variant (SNV) rs12640848 of the ENAM gene in students exposed to different concentrations of fluoride. Methods: A cross-sectional study was conducted on students exposed to high concentrations of fluoride in the city of Durango which were divided according to the severity of fluorosis and dental caries. Genotype determination was performed by DNA sequencing. The relationship between the severity of dental fluorosis and the allele distribution was determined by the Fisher’s exact and Kruskal–Wallis tests. Results: Seventy-one students were included for the sequencing. In the different allelic variations, for the normal genotype AA/TT, the control group presented 75%, for the AG/TC variation, 70.8% in the TF ≤ 4 group, 65% in TF ≥ 5, and 16.7% in TF = 0; with respect to GG/CC variation, 12.5% in TF ≤ 4, 22% in TF ≥ 5, and 8.3% in TF = 0 group (*p* = 0.000). Conclusion: The ENAM gene showed an association in the population exposed to different concentrations of fluoride.

## 1. Introduction

Dental enamel is a highly mineralized tissue and its formation, known as amelogenesis, is a process where the enamel-forming cells, the ameloblasts, deposit a thin, protein-rich matrix layer. These proteins control crystal growth, size, and thickness; subsequently, they are eliminated by proteases, resulting in crystal mineralization [1,2].

Ameloblasts mediate cellular processes during all stages of tooth enamel formation, including differentiation, cellular interaction, migration, and secretion of enamel matrix proteins, as well as maturation, in which different proteins that make up the extracellular matrix participate to achieve crystal growth and mineralization. The structural proteins amelogenin (AMELX), ameloblastin (AMBN), enamelin (ENAM), the proteinase matrix metalloproteinase-20 (MMP20, enamelysine), and the glycosylated serine protease kallikrein 4 (KLK4), among others, play important roles in tooth enamel formation [3,4].

Dental caries is one of the most prevalent chronic diseases, affecting the majority of children, adolescents, and adults around the world. Dental caries affects the mineralized tissue of the tooth, being a multifactorial disease induced by biological and environmental factors. Genetic association studies on caries have suggested that caries can be influenced by polymorphic variants in ameloblastin, amelogenin, enamelin, MMP20, tuftelin, and tuftelin interacting protein 11 [5,6].

Ingestion of excessive amounts of fluoride affects ameloblasts during different stages of enamel formation, resulting in different types of tissue defects, ranging from white opaque calcareous areas from subsurface hypomineralization to more severe defects [7]. The presence of hypomineralization, combined with the increased porosity of tooth enamel, causes the loss of important portions of the enamel structure, producing areas with hypoplasia that can cause fractures that deteriorate the appearance and functionality of the affected teeth [8].

Variation in the genes involved in amelogenesis can have an inherited, environmental, or multifactorial origin. Currently, studies have examined the genetic basis of tooth development disorders. These studies have focused on mutations in the genes encoding enamel matrix proteins and have found mutations in both structural proteins and proteases responsible for enamel matrix elimination and remodeling [9,10].

The ENAM gene encodes an enamelin protein from the enamel matrix and is composed of 1142 amino acids, with a signal peptide of 39 amino acids. This gene is essential for the formation of tooth enamel from developing crystals, which occurs before amelogenin expression. Its exact function is not yet fully known, although it is believed to be involved in the elongation of developing crystals that form perpendicular to the outer surface of the tooth, which contributes directly to the thickness of the enamel attached to the amelogenins and ameloblastines. The physical properties of enamel depend on the expression of ENAM and the amelogenin and ameloblastine genes [11,12,13].

Studies have reported that ENAM rs12640848 may be related to hypomineralization and dental caries, therefore it is important to study the variation in order to understand the molecular pathogenesis of tooth enamel formation, a stage with a greater susceptibility to fluoride overexposure. Thus, the aim of this study is to establish the presence of single nucleotide variant (SNV) rs12640848 and use DNA sequencing to determine its variation in the population exposed to different concentrations of fluoride.

## 2. Materials and Methods

### 2.1. Ethical Considerations

This study was reviewed and approved by the Ethics Committee of Metropolitan Autonomous University, Xochimilco, DCBS#3450491-CE.2018.009, and by the Ethics Committee at the Juarez University of Durango State EC-FO-UJED-01-14. The work was done according to the Declaration of Helsinki (1964), and the patients’ privacy data were protected.

### 2.2. Study Zone

In the City of Durango, high concentrations of fluoride in water (≥4 ppm) and endemic dental fluorosis have been reported [14], and in the State of Mexico, fluorosis has been reported as ≤0.3 ppm [15].

### 2.3. Study Population

A cross-sectional and experimental study was conducted on 11-year-old schoolchildren from a population of 105 students of both genders. An informed consent form was given for parental or guardian authorization. The inclusion criteria were the following: having lived since birth in the study area, having a permanent dentition, and the child’s voluntary participation in the study. Children who had undergone orthodontic treatment or had an oral health condition that would make it difficult to perform an appropriate oral or dental exam, and schoolchildren who refused to cooperate at the time of the exam, were excluded.

### 2.4. Sample Size

Of the 105 schoolchildren, only 82 gave informed consent and met the requirements to participate in the study. A survey was applied to obtain sociodemographic variables, and a clinical examination was carried out for the diagnosis of dental fluorosis and caries. Of this population, 71 schoolchildren underwent molecular analysis and sequencing; 11 schoolchildren were excluded due to an insufficient DNA sample.

### 2.5. Clinical Evaluation

Clinical evaluation was performed with consideration of the criteria established by the World Health Organization (WHO) [16]. The diagnosis and severity of dental fluorosis was determined according to the Thylstrup-Fejerskov (TF) index [17]. The study population exposed to high concentrations of fluoride (≥ 4 ppm) presented different phenotypes of dental fluorosis, therefore, the severity was divided into two groups: TF ≤ 4 (without loss of enamel surface) and TF ≥ 5 (with loss of enamel surface). A control group was included without dental fluorosis (TF = 0). The evaluation of fluorosis and dental caries was performed by two calibrated researchers at the intra- and interexaminer levels, obtaining Kappa values of ≥ 0.89.

### 2.6. DNA Extraction and Purification

After the clinical evaluation, samples were collected for DNA extraction by rubbing the buccal mucosa with a cytological brush to collect epithelial cells from each of the participants, and were then preserved in PBS buffer solution. A 1.5-mL Eppendorf tube was used to perform DNA extraction and purification, based on the method proposed by Aidar and Line [18]. The DNA obtained was quantified using a NanoDrop 2000c spectrophotometer (Thermo Scientific).

### 2.7. Primer Design

The Primer Blast NCBI program [19] was used to design the primers. Primers were designed specifically for sequencing the SNV selected in the ENAM gene (rs12640848), from which 400 bp products were obtained, with the variant located in the middle of the PCR product. Table 1 shows the primer characteristics.

### 2.8. PCR and DNA Sequencing

After amplifying the samples, the PCR products of 71 students were genotyped in duplicate to obtain 142 samples in both directions. A negative control containing only Taq polymerase, buffer, and primer was used without including the DNA sample for each PCR amplification reaction. The sequencing and presence of the SNV was confirmed by comparing sequences from both directions using the 400 bp custom sequencing method from Macrogen Inc., Korea. The allelic profile of the samples (chromatograms) was obtained through sequence-independent alignment in each subject, using the Chromas DNA sequencing software (Technelysium).

### 2.9. Statistical Analysis

Statistical analysis was performed using SPSS version 23 (SPSS, Inc., Chicago, IL). The variables were evaluated in univariate form for percentages and distributions. The significance of the differences in the allele distribution between TF ≤ 4, TF ≥ 5, and TF = 0 groups was tested using the nonparametric Kruskal–Wallis and Fisher’s exact tests, with a p-value of < 0.05 indicating a significant difference.

## 3. Results

The results show that from the total population, the control group without fluoride exposure presented more caries in comparison to the population exposed to high concentrations of fluoride, where 28.6% presented caries in TF < 4 group, 59.3% in the TF group ≥ 5, and the TF = 0 group (control) had the highest prevalence of schoolchildren with caries (81.5%).

For DNA analysis, Table 2 shows the result of the allelic frequencies of the ENAM gene, with 75% of the subjects in the TF = 0 category exhibiting the normal genotype AA/TT. The 12.5% of the subjects in the TF ≤ 4, 22% TF ≥ 5 and 8.3% TF = 0 category exhibited the GG/CC variant, presenting a different fluorosis phenotype.

Furthermore, differences were found in the three groups in the AG/TC variant (70.8% TF ≤ 4, 65% TF ≥ 5, 16.7% TF = 0 (*p* = 0.000)).

**Figure 1:**
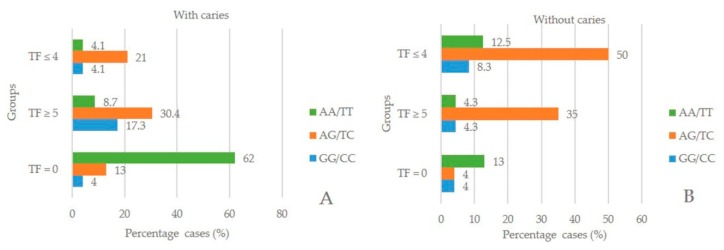
Frequency distribution of alleles in groups with different severity of fluorosis with, and without caries. (**A**) With caries. (**B**) Without caries.

Figure 1 shows the frequency distribution of the three study groups, with regard to the variation in alleles in schoolchildren with, and without, caries. Figure 1A shows the relationship of the alleles and caries, where the normal genotype AA/TT was present in 62% of the TF = 0 control group, and for the AG/TC variation, 21% was present in TF ≤ 4, 30.4% in TF ≥ 5, and 13% in TF = 0. In the GG/CC variation, it was found to be 4.1% in group TF ≤ 4, 17.3% in TF ≥ 5, and 4% in TF = 0. Figure 1B shows the frequency distribution of the three study groups regarding the relationship of alleles and without caries, in the genotype AA/TT: 12.5% in TF ≤ 4, 4.3% in TF ≥ 5, and 13% in TF = 0 was present. For the AG/TC variation, 50% was present in TF ≤ 4, 35% in TF ≥ 5, and 4% in TF = 0, while for the GG/CC variation, 8.3% in TF ≤ 4, 4.3% in TF ≥ 5, and 4% in TF = 0 presented differences between the three groups (*p* = 0.000).

**Figure 2:**
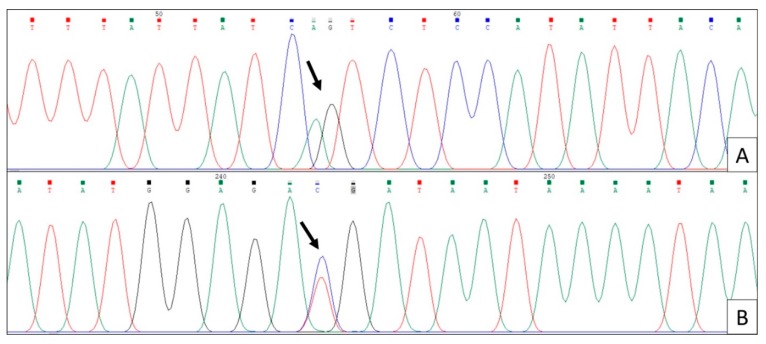
The chromatogram showing ENAM with the AG/TC variant alleles. (**A**) Forward direction. (**B**) Reverse direction.

Figure 2 shows the bidirectional chromatograms indicating the AG/TC alleles in a child from the study population from the TF ≤ 4 group presenting dental fluorosis and caries.

In line A of the forward direction, the A alleles are represented in green, and G alleles are represented in black. In line B of the reverse direction, with T alleles represented in red, and C alleles represented in blue with this sequencing, the genetic variability rs12640848 was identified.

## 4. Discussion

The results show that the control group of the population without fluoride exposure presented more caries in comparison to the population exposed to high concentrations of fluoride. These results agree with a study conducted by Molina–Frechero et al. in 2012 that assessed dental fluorosis and caries, where the population without exposure to fluoride presented more caries in comparison with the population exposed to fluoride that presented a low prevalence of dental caries [20].

Regarding the population that presented the allele variant AG/TC, TF ≤ 4 group, with a lower severity of dental fluorosis, presented a higher percentage of this variation, thus, it may be suggested that this variation plays an important role in association with the less affected phenotypes by fluorides. A study conducted by Shaffer et al., where the AG mutation was analyzed in the variant rs12640848 of ENAM among other genes, concluded the presence of interactions between the genes of the enamel matrix and fluoride exposures, mentioning that those which were exposed to fluorides had a higher amount of tooth decay in comparison with those which were not exposed to the sources of fluoride [21].

In the group with the highest severity of dental fluorosis, the GG/CC variant was predominant, with differences between the groups with and without dental fluorosis that may be explained by genetic factors, giving the presence of these mutations as a result. These results agree with the reported by Küchler et al., who demonstrated that some polymorphisms of AMELX, AMBN, ENAM, and TFIP11 genes are associated with susceptibility of dental fluorosis [22].

Regarding dental caries, the AA/TT alleles, that represent the normality of the association between bases, showed the highest percentage of dental caries in the control group, therefore, it may be considered that these are the most representative of the group without dental fluorosis. The variant AG/TC was present in a higher frequency in the schoolchildren with a lower severity of dental fluorosis. Otherwise, the variant GG/CC was predominant in the group with higher severity of dental fluorosis, where the enamel is more hypomineralized and presents areas with hypoplasia.

Regarding the schoolchildren without caries, AA/TT alleles were observed in a lower proportion in comparison to the schoolchildren with caries. In the variant AG/TC, some differences were found according to the severity of dental fluorosis, with a higher proportion of schoolchildren without caries in the TF ≤ 4 group. This finding is not consistent with the study made by Borilova et al., which did not observe significant differences in allelic or genotypic frequencies between children with and without caries; that study considered the presence and absence of caries and the ENAM variant (rs12640848) [23].

In a study developed by Negre at al., the presence of an association between dental caries and affected surfaces by hypomineralization were found, concluding that the hypomineralized enamel has more susceptibility to development of this type of lesions [24].

This variant has been widely studied in relation to dental caries. Likewise, in our study we found that this gene may predict the developing of these lesions, such as that reported in other studies where the presence of the ENAM variant rs12640848 in the population may predict the development of caries; this finding is consistent with the study made by Gerreth et al., which reported the association of the ENAM gene and the susceptibility to caries in primary teeth in children [25], as well as that reported by Devang et al., which found a significant association between other variants of the ENAM variant rs7671281, and susceptibility to dental caries [26].

However, some interactions or factors may be related to genetic variability, such as genetic background or environmental factors [27]. This could result in variable genetic effects observed in schoolchildren. Thus, it is important to perform further studies that include whole families in order to confirm the mutated alleles.

The importance of the current research relies on the lack of studies that focus on enamel development genes in the context of high levels of fluoride, which could have an important role in the susceptibility to dental fluorosis. Thus, our results may contribute to the genotypic identification of *ENAM* as a protective factor against dental caries and to the molecular characterization of the dental enamel at the exposure to high concentrations of fluorides. 

One limitation of this study was the sample size. Thus, these analyses should be replicated in different populations with larger sample sizes. In addition, related genes should be investigated to clarify the relationship between this polymorphism, and susceptibility to dental fluorosis and caries. Our data support the hypothesis that the ENAM gene rs12640848 can be a genetic component associated with different concentrations of fluoride.

## 5. Conclusions

The ENAM gene plays an important role in tooth enamel development and the etiology of dental fluorosis. Our results show that the effects of fluoride exposure develop different phenotypes of dental fluorosis, and the presence of the variant rs12640848 differs between the different phenotypes and the group without fluoride exposure, which can be protective, and can avoid the effects of mutant alleles. Thus, it is important to conduct further studies in larger populations on the pathogenesis of dental fluorosis.

## Figures and Tables

**Table 1:** Primers designed for *ENAM variant rs12640848*.

Gene	Name	Chromosome	SNV	OMIM	Product Size
*ENAM*	Enamelin	Location: 4q13.3	rs12640848	* 60655	
Direction	Primer 5′-3′	TM	GC	Long	
Forward	AAGTACCAGGCACTGAGCTG	59.68	55.00	20	400
Reverse	CTTTCCTTTAATCTTGCTTCTGCAC	59.14	40.00	25	

*: Known inheritance mode.

**Table 2:** Distribution of number and percentage of alleles by groups according to different exposure of fluoride concentrations.

ENAM rs12640848	≥4 ppm				≤0.3 ppm		*p*-Value
	TF ≤ 4		TF ≥ 5		TF = 0		
Alleles Fw/Re	*n*	%	*n*	%	*n*	%	
	AA/TT						
	4	16.7	3	13	18	75	0.000
	AG/TC						
	17	70.8	15	65	4	16.7	0.000
	GG/CC						
	3	12.5	5	22	2	8.3	0.000
Total	24 Samples		23 Samples		24 Samples		

TF ≤ 4 lower severity of dental fluorosis, TF ≥ 5 TF higher severity of dental fluorosis, TF = 0 without dental fluorosis.
